# Strategies to mitigate student resistance to active learning

**DOI:** 10.1186/s40594-018-0102-y

**Published:** 2018-03-12

**Authors:** Sneha Tharayil, Maura Borrego, Michael Prince, Kevin A. Nguyen, Prateek Shekhar, Cynthia J. Finelli, Cynthia Waters

**Affiliations:** 1 University of Texas at Austin, Austin, TX USA; 2 Bucknell University, Lewisburg, USA; 3 University of Michigan, Ann Arbor, USA; 4 North Carolina Agriculture and Technical State University, Greensboro, USA

**Keywords:** Active learning, Student resistance, Instructional strategies, Undergraduate engineering

## Abstract

**Background:**

Research has shown that active learning promotes student learning and increases retention rates of STEM undergraduates. Yet, instructors are reluctant to change their teaching approaches for several reasons, including a fear of student resistance to active learning. This paper addresses this issue by building on our prior work which demonstrates that certain instructor strategies can positively influence student responses to active learning. We present an analysis of interview data from 17 engineering professors across the USA about the ways they use strategies to reduce student resistance to active learning in their undergraduate engineering courses.

**Results:**

Our data reveal that instructor strategies for reducing student resistance generally fall within two broad types: explanation and facilitation strategies. Explanation strategies consist of the following: (a) explain the purpose, (b) explain course expectations, and (c) explain activity expectations. Facilitation strategies include the following: (a) approach non-participants, (b) assume an encouraging demeanor, (c) grade on participation, (d) walk around the room, (e) invite questions, (f) develop a routine, (g) design activities for participation, and (h) use incremental steps. Four of the strategies emerged from our analysis and were previously unstudied in the context of student resistance.

**Conclusions:**

The findings of this study have practical implications for instructors wishing to implement active learning. There is a variety of strategies to reduce student resistance to active learning, and there are multiple successful ways to implement the strategies. Importantly, effective use of strategies requires some degree of intentional course planning. These strategies should be considered as a starting point for instructors seeking to better incorporate the use of active learning strategies into their undergraduate engineering classrooms.

## Background

Extensive research has shown that active learning strategies are generally more effective than traditional lecture for promoting a wide range of desirable educational outcomes, including increased student learning and better retention in STEM programs (Freeman et al. 2014; Michael 2006; Prince 2004; Prince & Felder 2007; Lund and Stains 2015). However, the adoption of these evidence-based instructional strategies into actual classroom practice has been slow (National Research Council 2012; American Society of Engineering Education 2012; Friedrich et al. 2009; Hora et al. 2012). Past surveys of STEM instructors indicate a number of specific barriers that hinder their use of active learning strategies (Shadle et al. 2017; Lund and Stains 2015 Dancy and Henderson 2012; Finelli, Daly, & Richardson 2014; Froyd, Borrego, Cutler, Henderson, & Prince 2013; Henderson and Dancy 2009; Prince, Borrego, Henderson, Cutler, & Froyd 2013). These barriers include concerns about (a) the effectiveness of these new methods, (b) preparation time, (c) the class time required to implement active learning and instructors’ consequent ability to cover the syllabus, and (d) student resistance, which includes any number of possible negative responses to the new teaching methods.

While most of these concerns have been sufficiently addressed through the existing literature (in addition to the previously cited resources, see also Felder 1992, 1994; Felder and Brent 2009), relatively little research addresses student resistance and how to mitigate it. Although much of the published literature suggests that students often respond positively to active learning strategies (Arce 1994; Armbruster et al. 2009; Carlson and Winquist 2011; Hoffman 2001; Leckie 2001; Oakley et al. 2007; O’Brocta and Swigart 2013; Reddy 2000; Richardson and Birge 1995), there are counterbalancing studies which show mixed student responses (Bacon et al. 1999; Brent and Felder 2009; Goodwin et al. 1991; Hall et al. 2002; Kvam 2000; Rangachari 1991; Wilke 2003) or negative student responses (Lake 2001; Yadav et al. 2011). While few (if any) prior studies empirically identify strategies instructors can use to mitigate student resistance to active learning, some studies include advice on how to reduce resistance. For example, Yadav et al. (2011) suggest that in implementing active learning, instructors should acknowledge the challenges of the new approach, provide students with feedback and support throughout the process (also suggested by Bentley et al. 2011), and solicit and act on student feedback about the activities. Based on their experiences implementing workbooks in a statistics course, Carlson and Winquist (2011) recommend ramping up slowly. Multiple authors recommend clearly explaining the purpose and expectations of the activity (Bacon et al. 1999; Strobel and van Barneveld 2009; Wilke 2003) and aligning activities with other course assessments (Bentley et al. 2011; Donohue and Richards 2009). Most of these recommendations, however, are anecdotal and do not have much empirical support. There is therefore a need to more closely and empirically examine the practical ways with which STEM instructors can bolster active learning in their classrooms.

In our previous research on student resistance, we conducted observations in several introductory engineering classrooms (Shekhar et al., 2015) which led to development of a survey instrument to study student resistance to active learning, Student Response to Instructional Practices (StRIP) (DeMonbrun et al., 2017). Based on the observations, we built on the strategies listed above that instructors could use to reduce student resistance. This work included a framework for instructor strategies along the lines of explanation and facilitation (DeMonbrun et al., 2017; Nguyen et al., 2017b). *Explanation* strategies describe the purpose of the in-class activity. *Facilitation* strategies promote engagement and keep the activity running smoothly once it has already begun.

Preliminary testing with four active learning courses and 179 students highlighted the lack of student resistance to active learning (Nguyen et al., 2017b). The linear regression analysis used students’ expectation and experiences of different types of teaching methods as well as students’ perceptions of instructor strategies to predict how students responded to active learning. We found that students’ report of the frequency of instructor strategies was both a significant and strong predictor for how students responded to active learning. The instructor strategies were explanation and facilitation, as described previously. Student responses to active learning included the value they placed on the activities (final model *R*^2^ = 0.44), their positivity toward the activities (*R*^2^ = 0.56), their participation in the activities (*R*^2^ = 0.12), and overall course evaluation (*R*^2^ = 0.58). In sum, our linear regression models highlighted how instructor strategies significantly and positively predicted higher student value, positivity, and course evaluations.

Our full-scale study of 1051 engineering students in 18 courses (the same ones in the present study) found that explanation and facilitation instructor strategies were again significant predictors of how students responded to active learning (Finelli, C. J., Nguyen, K., DeMonbrun, R.M., Borrego, M., Prince, M., Henderson, C., Husman, J: Reducing student resistance to active learning: Strategies for instructors, forthcoming). Explanation and facilitation were significantly correlated with students’ value, positivity, participation, distraction, and course evaluation. Our linear regression suggested that instructor strategies as well as students’ value and positivity are significant positive predictors of students’ participation (final model *R*^2^ = 0.32) and course evaluation (*R*^2^ = 0.32) and negative predictors for students’ distraction (*R*^2^ = 0.20). The robustness of instructor strategies as predictors in both our preliminary and full-scale regression modeling suggests that accounting for students’ perceptions of the frequency of instructor strategies seems to be both the most actionable and positive way to affect student resistance and response to active learning (Finelli, C. J., Nguyen, K., DeMonbrun, R.M., Borrego, M., Prince, M., Henderson, C., Husman, J: Reducing student resistance to active learning: Strategies for instructors, forthcoming).

Building on these prior findings, the present work seeks to examine how engineering instructors employ explanation and facilitation strategies by qualitatively analyzing instructor interviews associated with the courses discussed in the studies above. More specifically, this paper investigates the following research questions:

- How are engineering instructors using the explanation and facilitation strategies that previous analyses have identified to be effective in reducing student resistance to active learning?
- What other additional strategies are engineering instructors using to reduce student resistance to active learning, and how are they using them?

### Qualitative methods

#### Overview

The interview data presented here are part of a larger study, which includes classroom observations, student surveys collected at the three points in the semester, and instructor surveys collected at the beginning and end of the semester. We triangulated data sources from students, instructors, and our own observations and found consistency regarding what happened in class in type and frequency of active learning activities, instructor strategies for reducing resistance, and student resistance and participation (DeMonbrun et al., 2017, Shekhar et al. 2015). Our prior analyses found that explanation and facilitation instructor strategies were effective in reducing student resistance and increasing participation (Nguyen 2017b). This paper focuses on the instructor interviews we conducted at the end of the semester. We applied a thematic analysis (Boyatzis 1998) to the interview data to understand how instructors utilized strategies to reduce resistance in terms of explanation and facilitation strategies.

#### Sampling and participants

Employing both convenience and purposive sampling, we posted email solicitations on relevant listservs to recruit engineering instructors who self-identified as frequent practitioners of active learning. Although self-selected, most, if not all, of the participant instructors seemed to indicate in their interviews that they had prior experiences using active learning teaching methods. The sample of experienced and confident engineering instructors provided opportunities to examine their rationale and implementation of continued active learning instruction. Instructors also agreed to administer the StRIP survey to their students at three times during the term, to complete the instructor version of the StRIP instrument at the beginning and end of the term and to participate in an end-of-term instructor interview. We conducted interviews in the Fall 2015 and Spring 2016 semesters. We were able to gather complete interview data from 17 of the original 18 instructors. These 17 interviews comprised the raw data for the present study.

Our sample includes a broad mix of instructor genders, ranks, course disciplines, class sizes, and institution types (see Table 1). One instructor was black or of African descent; all others were white or of European descent. All the courses were lecture-based (as opposed to lab-based), and were either first or second year core courses for undergraduate engineering majors. Some of the larger courses included instructional staff such as teaching assistants (TAs) that helped the main instructor. Teaching methods in the sampled courses included not only active learning and non-traditional instructional practices but traditional practices (e.g., lecturing) as well (Nguyen et al., 2017a; Nguyen et al., 2017b). Attempts to more specifically quantify and qualify the types and extent to which active learning occurred in each course proved to be more difficult. Our previous studies showed that parsing out active learning features within a course can be problematic due to several conflating factors (Nguyen et al., 2017c), even when using theoretical frameworks like the Interactive-Constructive-Active-Passive (ICAP) framework proposed by Chi and Wylie (2014).

**Table 1:** Course demographics

Pseudonym	Instructor gender	Instructor rank	Course discipline^1^	No. of students	Institution type	Carnegie classification^2^
Betsy	F	Sr. lecturer	CBME	97	Private	M1
Esther	F	Assoc. prof.	CIVIL	51	Public	R2
Emily	F	Asst. prof.	CBME	131	Public	R1
Megan	F	Asst. prof.	CBME	79	Public	R2
Michelle	F	Asst. prof.	INTRO	123	Private	M1
Patricia	F	Assoc. prof.	IDISC	74	Private	R2
Samantha	F	Asst. prof.	INTRO	28	Public	BACC
Tracey	F	Assoc. prof.	CBME	41	Public	R1
Benjamin	M	Asst. prof.	INTRO	286	Public	M1
Chris	M	Assoc. prof.	MAT	28	Public	BACC
Daniel	M	Full prof.	CBME	119	Public	R1
David	M	Full prof.	ME	45	Public	M1
George	M	Asst. prof.	INTRO	35	Public	R1
Justin	M	Assoc. prof.	ME	36	Public	M1
Matthew	M	Adjunct prof.	EECS	226	Public	R1
Peter	M	Asst. prof.	INTRO	51	Public	R1
Timothy	M	Asst. prof.	EECS	11	Private	M3
William	M	Assoc. prof.	INTRO	140	Private	R2

^1^Course disciplines: *CBME* Chemical/Biomedical Engineering, *CIVIL* Civil and Environmental Engineering, *DESIGN* Design, *EECS* Electrical Engineering/Computer Science, *IDISC* Interdisciplinary, *INTRO* Introduction to Engineering, *MAT* Materials Science and Engineering, *ME* Mechanical Engineering

^2^Carnegie classifications: *R1* Doctoral Universities: Highest Research Activity, *R2* Doctoral Universities: Higher Research Activity; *M1* Master’s Colleges and Universities: Larger Programs, *M3* Master’s Colleges and Universities: Smaller Programs, *BACC* Baccalaureate Colleges: Arts & Sciences Focus combined with Baccalaureate Colleges: Diverse Fields

#### Interview protocol

We utilized an individualized, semi-structured interview protocol. The protocol included an introduction to our project, followed by questions about the types of active learning the instructor used over the term, how that compared to previous courses, and whether the instructor believed the active learning “worked.” The remainder of the interview revolved around a customized report that summarized student and instructor survey responses for the course. The protocol included sections for the instructor to:

- Compare the frequency of active learning instruction reported by students and instructors
- Reflect on the purpose of using the active learning in-class activities
- Compare the instructor’s beginning-of-term prediction of how students would respond to active learning to students’ actual end-of-term response
- Compare student and instructor reports of frequency of strategies to reduce resistance
- Consider possible explanations for student resistance
- Reflect on characteristics of students’ ideal type of instruction
- Consider ways the instructor might approach the class differently to reduce resistance in light of the feedback
- Offer suggestions for improving the surveys and report

Three research team members piloted and revised the interview protocol from Spring 2014 through Spring 2015 on six instructors from four different institutions. For the full analysis, five research team members conducted the interviews, primarily on Skype™, and the interviews ranged from 28 to 80 min (mean = 49 min, standard deviation = 15 min). In sum, seven research team members piloted or conducted the interviews. Student survey results are reported elsewhere (Finelli, C. J., Nguyen, K., DeMonbrun, R.M., Borrego, M., Prince, M., Henderson, C., Husman, J: Reducing student resistance to active learning: Strategies for instructors, forthcoming; Nguyen et al., 2017a).

#### Systematic thematic coding of interview data

We prepared the interview data for analysis by transcribing all audio files, removing identifying information, and cleaning the data for clarity. Two members of our research team then read all 17 transcripts and highlighted all passages related to instructor strategies and reducing student resistance. A third member of the research team exported these passages into smaller excerpts for analysis in a Google Sheets™ spreadsheet. Four other members then coded the excerpts. First, the four members coded and discussed the excerpts from the same instructor to norm the coding process and interpretation of the codes. Next, the four coders divided the remaining instructors and applied an initial set of codes based on instructor strategy items on StRIP survey (Table 2). Then, the coders met and agreed on additional strategies not included in the StRIP instrument and added codes. Finally, the four coders divided the codes into related clusters to finalize the coding. They met regularly to reach agreement on the final set of codes and their definitions (Table 2).

**Table 2:** Coding scheme with definitions

Original strategies taken from StRIP survey	Final code	Description of final code
*Explanation strategies*		
Clearly explained the purpose of the activities.	Explain the purpose.	Providing students with a rationale for using active learning in the classroom by explaining how the activities relate to their learning, connecting the activities with course topics, discussing their relevance 7to industry, etc.
Discussed how the activities related to my learning.		
(Emergent strategy)	Explain course expectations.	Communicating overall course expectations for student participation at the beginning of the semester
Clearly explained what I was expected to do for the activities.	Explain activity expectations.	Providing explicit instructions about what students are expected to do for a specific active learning exercise
*Facilitation strategies*		
Confronted students who were not participating in the activities	Approach non-participants.	Confronting students who are not participating in activities by physically approaching them, calling on them during more structured lecture, etc.
Encouraged students to engage with the activities through his/her demeanor.	Assume an encouraging demeanor.	Establishing verbal or non-verbal cues such as setting a tone for risk taking, caring about students’ success, encouraging responses by using uncomfortable silences, etc.
(Emergent strategy)	Grade on participation.	Using points or grades to encourage participation
Walked around the room to assist me or my group with the activity, if needed.	Walk around the room.	Walking around the room during active learning instruction
Solicited my feedback or that of other students about the activities.	Solicit student feedback.	Encouraging students to provide feedback about an in-class activity
Invited students to ask questions about the activities.	Invite questions.	Prompting students to ask questions about an in-class activity during that activity
(Emergent strategy)	Develop a routine.	Establishing an “active learning” routine by having a standard type of “bell work,” using a systematic approach to interact with students during an activity, regularly calling on students by name, etc.
(Emergent strategy)	Design activities for participation.	Structuring an activity so that students will be more likely to engage in active learning by creating student groups, reframing tasks, etc.
(Emergent strategy)	Use incremental activities.	Integrating support mechanisms to help students accomplish more complex tasks by giving hints, decomposing a problem into parts, etc.

## Findings

The interview data showed that, on the whole, the instructors used a variety of explanation and facilitation strategies. Table 3 depicts which instructors indicated use of each strategy. The following paragraphs explain each category (i.e., explanation and facilitation) and strategy in detail and how the instructors used them. Four potentially new strategies emerged from the data analysis; all were facilitation strategies. These are described at the end of this section.

**Table 3:** Strategy use by instructors

Instructor pseudonym	Explain the purpose	Explain course expectations	Explain activity expectations	Walk around the room	Approach non-participants	Assume an encouraging demeanor	Invite questions	Solicit student feedback	Develop a routine	Grade on participation	Design activities for participation	Use incremental steps
Betsy	x	x		x	x	x	x		x	x	x	x
Emily	x	x		x		x		x				
Esther	x	x	x	x	x		x	x		x		
Megan				x	x		x	x	x			
Michelle	x	x		x	x	x	x	x	x	x	x	x
Patricia		x										
Samantha				x	x		x					
Tracey	x	x		x	x		x	x	x			
Benjamin	x	x		x	x		x					
Chris	x	x	x	x	x		x	x			x	
Daniel	x	x		x		x	x	x			x	
David	x			x	x			x			x	
George	x	x			x	x	x		x		x	
Justin				x	x					x		x
Peter	x	x	x	x	x	x	x	x			x	
Timothy	x	x	x	x	x		x		x	x		x

### Explanation strategies

Explanation strategies involved the instructor explaining the components of the course, activities, or the rationale for course activities or other elements. These strategies were clarifying in nature or elucidated to students the instructional decisions and rationales behind course structure and activities. They attempted to help students either meaningfully and successfully engage in an active learning activity or understand how the activity helps meet course-learning goals. These explanation strategies encompassed three different approaches: (1) explain the purpose, (2) explain course expectations, and (3) explain activity expectations.

#### Explain the purpose

Explaining the purpose of the activities provided students with a rationale behind the use of active learning in the classroom. One facet of explaining the purpose involved explaining to students how the activities related to their learning. For instance, Michelle explained, “every class period I make connections between what I’m about to do and whether we’re working on procedural knowledge or we’re working on conceptual knowledge or we’re working on meta-cognitive knowledge.” Similarly, Betsy explicitly explained how the activities would be helpful in exams. She continued, “I do try to remind them that I’m not going to waste their time on busywork. That anything I ask them to do will be beneficial to them as learners.”

In contrast, Daniel followed a more discussion-based approach and underscored the importance of engaging students in reflection about their learning. He explained,

I think explaining the purpose of the activity is critical, and explaining how [activities] relate to learning. […] explaining the purpose of the activity and reflecting on that. I ask my students a lot about how they are making meaning of their experiences. That’s part of what I do for instruction. I mean literally every week they are asked to reflect on what’s going on and they communicate that.

Instructors also explained the value of the activities in terms of their relevance to industry, connection with course topics, and importance in completing homework and exams. For example, Esther noted, “I was able to explain to them in the context of what goes on in industry, and I did that to explain why I give them open-ended questions.” Tracey simply explained the connection between the activities and various course topics by announcing to students “this is the main topic for the day, these are the critical equations or concepts that you’re applying.”

#### Explain course expectations

Instructors also frequently explained course expectations as a strategy to reduce student resistance. They emphasized on the importance of communicating course expectations at the beginning of the semester. For example, Tracey explained:

That first day I hit that pretty hard. They come in and they look at the room and they think about what is different [about the SCALE-UP configuration] and we talk about that. And then I just go through and explain what flipping is and why we are doing it this way. So that’s probably something when I was working with our Center for Teaching to do the flip, that was the aspect that they recommended the most is that you need to hit this right off the bat.

Similarly, Patricia mentioned, “setting the expectations in the first few weeks by explaining to students what they were expected to do.” William followed a more formal approach wherein he delineates these expectations clearly as a part of his syllabus:

I’ve attached an extra page to the syllabus that explains to them what they’re getting into. It tells them that I want to work with them to make it easier for them, but it requires them engaging and participating with me. I especially can’t help them if they don’t come to class. At the end of the page, it asks them if they’re in? Yes? No?

Like “explain the purpose,” instructors used multiple methods to explain course expectations from verbal explanations to discussion to addendums in syllabi.

#### Explain activity expectations

Instructors also reported explaining activity expectations as a helpful strategy. This involved providing detailed instructions of how students should perform the assigned tasks. For example, while using case studies to actively engage students, Esther told her students which case studies have one correct answer and which have “multiple views.” Chris reported that he explained what students were required to do by engaging them in a conversation about the purpose of discussion. In a similar vein, George reported that he planned to add more structure to his exercises in future course offerings to provide clearer instructions to students. Thus, as comments such as these demonstrate, instructors endorsed the importance of explaining activity expectations as a means to help students respond positively to active learning.

### Facilitation strategies

Facilitation strategies are employed by instructors both before and during the implementation of active learning activities to reduce student resistance. These strategies include (1) walk around the room, (2) approach non-participating students, (3) assume an encouraging demeanor, (4) invite questions, (5) solicit student feedback, (6) develop a routine, (7) grade on participation, (8) design activities for participation, and (9) use incremental steps. While the first five strategies were identified in the original StRIP survey, the latter four strategies emerged as recurring themes across the interview data.

#### Walk around the room

Among the facilitation strategies, walking around the room seemed to be the most effective strategy and certainly the one described in greatest detail. As Justin clarified, this strategy is “not just walk around, but walk around and stop” to talk with students about their work. He continued, “I think it may lower the stakes a little bit for the students so it makes it more okay to not know, or to be wrong, or to try something.” Michelle explained the rationale this way:

Walking around is the whole reason why active learning works. If [you let] your students loose on something and then you’re up at the front of the room not engaging with them at all you’ve lost that opportunity to figure out what students are doing. And you’re not making yourself accessible to ask questions or anything like that. So, the whole reason that active learning works is because you’re allowing them to work at their pace and struggle and things like that. But you want to be there when they run into a roadblock, and a lot of times they won’t raise their hand if they run into a roadblock. You can only see that they’ve done that by the fact that they haven’t make any progress yet. You have to go around and look.

Instructors described situations in which students asked them questions or they were able to identify students’ mistakes as a result of walking around the classroom. Megan commented, “the students don’t hesitate to flag any of us down if they have a question […] I feel like there’s good buy in and then there’s also a lot of mechanisms to give students feedback and help along the way.” David explained, “I just walk around and sometimes I’ll see a mistake. A clear mistake. […] If I see a mistake we’ll talk about it right then.” Similarly, Tracey explained that walking around allowed her and her TAs to see student work and “identify that they’re going along the wrong path. We go immediately up to them and help them get back on the right track.”

Another important benefit of walking around is that it helped keep students on task, reducing the need for more direct confrontation. For example, Benjamin commented that his StRIP student survey results indicated that he confronted students seldom because “It’s more like walking around, and then you go to a team and you ask them, ‘How are you doing? What do you think about that?’” Thus, as these instructor comments indicate, walking around served multiple purposes.

Some instructors, however, commented on hindrances to implementing this strategy effectively. For example, Megan, Samantha, Daniel, and Betsy all mentioned the layouts of their classrooms as not being ideal for accessing all students individually when walking around. As Daniel explained, “the seats don’t turn, and it’s a big lecture hall. It’s really hard for me as an instructor to get access to some students. Just physically they can sit in places that I have no way to get close to them.” Megan explained that some of the best-equipped classrooms are in high demand and only hold 60 students, which might require her to teach two sections of her course if scheduled there. Nonetheless, the instructors emphasized walking around as an important strategy and did what they could in the spaces they were provided.

#### Approach non-participants

One of the survey items was “Confronted students who were not participating in the activities.” Even though students reported some frequency for use of this strategy, many instructors still expressed discomfort with such strong language. For example, Esther stated, “I don’t confront them because students do not like to be confronted amongst their peers.” Betsy said, “I’m afraid that they'll feel put on the spot when they don’t want to be.” David explained that he does not want to put students on the spot because he remembers how badly that felt when he was a student.

Several instructors instead described more gentle ways of approaching students who do not appear to be working on the assigned activity. Michelle explained, “if I’m walking around during problem solving, and I see that a student doesn’t have anything written down on their page yet I might start by just asking ‘so are you thinking or are you stuck?’” William was more direct: “I would walk right up to them as they are staring into their smartphones, and I’d ask them a question about what we’re doing.”

Instructors seemed to have mixed feelings about calling on students during more formal report-back or lecture time. While Peter explained, “I never ever, ever call on students.” Timothy, George, and William lamented that although there are drawbacks to calling on students, it is a useful tool for engaging students. Timothy elaborated:

I can understand why students don’t want to be called on personally, and I'm pretty uncomfortable calling students by name in a class environment. But I think it’s important because unless I am calling on everyone, the conversation usually degrades to two or three people who like to talk to me or like to talk to the class. So getting multiple people talking in the class is an essential goal for me and right now that's one of my best tools to accomplish that.

Chris and Betsy were strategic in calling on students they suspected were distracted. Chris said, “I pay attention to who I am calling on and typically have a reason why I’m calling on them. And part of that is for me to monitor whether or not they’re actually engaged as opposed to doing something else.”

Other instructors described alternatives to confronting students or ways to lower the anxiety of responding when called on. Michelle assigned groups and seats for the entire semester so students get to know each other and are invested in others’ success, while Peter assigned project teams and had students discuss with their neighbors before asking for volunteers. Benjamin always called on teams instead of individuals. Tracey offered whiteboard markers to students who seem disengaged and invited them to work on the next step in group problem-solving. Esther and Peter found time to talk with teams or individuals privately to understand why students are not participating and discuss the implications of non-participation. On the other hand, some instructors, like Justin and David, mentioned forgoing approaching non-participating students if they make it clear they do not want to participate after repeated attempts.

#### Assume an encouraging demeanor

A few instructors discussed encouraging participation through their demeanor. For example, some described setting a tone that helps students be more comfortable with asking questions and the possibility of being wrong. Michelle said, “I do try to give a sense of safety, like if I call on you and you’re not ready yet that’s OK.” Similarly, William explained, “I think it’s good for them to know that I am also still here and they can come to me if they need any help.” Emily and George mentioned developing rapport with students on the first day of class. George and Michelle shared their passion for teaching with their students, along with Betsy, who said, “I try really hard to connect to the students and make them feel that I’m there for that. I really work hard for my rapport with the students. I feel like I try to respect them. […] I really try hard to make them understand that I really care how they do in the course.”

Instructors also leveraged this demeanor to prompt student engagement. For example, George explained how he used silence to encourage students to answer his questions: “I’m going to stand here until you guys are ready. I’m not afraid of silence.” George not only assumed silence to create space for student dialog, but he called attention to it to show the intentionality behind his silence. Thus, assuming an encouraging demeanor need not only establish an approachable rapport with students, but it can also be purposefully conveyed to elicit specific student behaviors.

#### Invite questions

One strategy that was consistently identified as being important among several instructors was “inviting questions.” Multiple instructors explained how encouraging student questions helped establish an encouraging climate for active learning in the classroom where students feel like their voices are heard and valued. Daniel said inviting student questions and cultivating an “interactive and responsive and generative” environment is crucial. Tracey similarly commented that it is important to create an environment in which “it’s okay to make mistakes, it’s okay to ask questions, that you’re better off asking questions now than getting a bad grade in an exam cause [sic] you don’t understand.” George discussed how it was important to still welcome student questions even when those questions may seem bizarre.

Instructors also commented on how encouraging student questions actually facilitated instruction in other ways. For example, George noted that much of his lecturing occurred while answering student questions. Esther also used her prompting of student questioning to coach students into different ways of thinking about the content: “I give them scenarios and I ask them to give me […] the type of questions you feel a contractor may ask regarding this scenario.” Betsy, on the other hand, explained how she formatively assesses her students’ understanding based on the type of questions she receives (and does not) from them.

Given the importance of student questions, several instructors also identified different techniques they use to invite students to ask questions. For example, Chris scaffolded students into asking questions by prompting them to “think of two questions on material you don’t understand or material [you] think would be [a] really good test question.” Betsy used wait time to elicit student questions, “I’m a big, big pusher of ‘are there any questions’ and I actually stop and wait, really leaving room for them to fill in the void.” Samantha and Michelle both used physical proximity to prompt and encourage student questions. Samantha said students “won’t ask questions […] unless I come over and say, ‘Hey, how’s it going?’ They’ll say, ‘How do you do this one?’ That [strategy], probably is the most powerful thing that works for me” because it communicates to students “Hey, we are here. I exist. Please ask me questions.” Michelle also designed her activities so that they forced students to ask questions. She described one such activity, “there is one particular assignment, a single one out of the thirty or forty that I give, where I intentionally don’t give them enough instructions, where I really want to see them struggle with it and ask questions.” As illustrated by this wide array of examples, inviting students to ask questions was identified by the instructors as an important strategy, and instructors often found creative ways to employ it.

#### Solicit student feedback

Some of the instructors also identified soliciting student feedback about the activities as being important for reducing further student resistance. For example, Peter explains how he has used surveys at the muddiest point to collect feedback from students:

At the end of the project, I always ask students, “Was there anything you would like me to change? […] This is something I am asking you to do and there is no credit for it but it’s because of the feedback that other students from previous sections that they have given me, that’s why the class is the way it is now. So it’s all about you continuing to give me more feedback so that I can continue to evolve this course.”

The instructors described ways of soliciting student feedback both explicitly and implicitly. Daniel simply asked students directly for feedback. Michelle, on the other hand, described more covert ways she obtained feedback from her students in addition to explicitly asking for it,

A lot of that is not done in a way that students would notice. I am looking at facial expression. I’m looking at turn-in rates. I’m looking at things that the students wouldn’t necessarily know that I’m doing but I do explicitly request feedback. Usually three or four times throughout the semester.

Similarly, Tracey explained how student feedback can be instrumental in guiding students to overcome some of their initial struggles when engaging in active learning activities:

Usually that first week it’s pretty rough and I just try to be really encouraging and say ‘it’s okay, come next week. It’s going to be a lot better.’ […] What isn't working so well that first week? […] Let’s work on these aspects and then by the end of second week, it’s definitely is a lot better after we have had multiple discussions and pointed out where we can improve.

It seems that by carefully attending to both explicit and implicit feedback provided by students, instructors use this feedback not only to tailor and improve their own instruction, but also to help temper student resistance to active learning by pinpointing the challenges students might experience while attempting an active learning activity and helping them overcome them.

#### Develop a routine

One potentially new strategy that emerged from the interviews was establishing a routine early in the term so that students expect active learning. Tracey explained the benefits of setting students’ expectations, saving time, and reducing anxiety. “They know as soon as they come in to expect that piece of paper on their desk, start working on that and then we're going to go into the group activity. So the transitions get quicker and quicker.” She had noticed that “It takes time for them to get used to how they should respond and behave.” Finally, she explained that routine reduces the “nervousness” that students can have about unfamiliar activities and “sets them at ease.” Similarly, Betsy “had the students working on problems in groups pretty much once a week.” Michelle explained how she also established a routine for calling on students, “something that I try to do in the first couple weeks is make sure I’ve called on everyone and in that first couple weeks it really sets the tone that when you come into class I’m going to engage with you. Be ready.” George used routines for discussion that he implemented from the very start of the semester to help students get comfortable interacting with each other. As expressed through these instructor reflections and those of others, establishing a routine with activities early in the semester seemed to set the tone and the expectation for students to engage during class. Furthermore, consistency fostered by the routine can reduce anxiety and eventually save some time.

#### Grade on participation

Grading emerged as another previously unstudied strategy for reducing student resistance. Three instructors used grades to encourage participation. Esther and Michelle sometimes awarded credit for completing an activity. Michelle also included a group grade on exams to encourage her group members to support each other’s learning.

While some instructors did incorporate participation into their grading schemes, others expressed some hesitation. Timothy, for example, attempted an explanation: “Maybe it’s just the authority of the professor that makes them think …it’s going to impact their grades somehow if they don’t. … One of the things I would say if the student was not engaging was a reminder that the material that you were doing in this activity could be in a future quiz.” Although Betsy occasionally assigned points for attendance, she distinguished participation from true engagement: “I try not to grade for participation because I don’t want students to feel like they can come and be passive and just sit, but they actually have to be active learners.” In sum, most of the instructors we interviewed did not directly use grading to encourage participation in active learning, although some instructors have found this approach to be successful.

#### Design activities for participation

Another strategy that emerged in the interview data was “design activities to encourage participation.” This strategy refers to the instructor’s thoughtful structuring of activities that demand an active role on the part of the student. This conscientious prior planning may involve considering various aspects of the activity including the task(s) itself, the cognitive demand, questioning strategies, and the group dynamics.

Chris discussed two ways he promoted participation. The first was to ask open-ended questions. “I very often do not ask questions that the answer is going to be something that’s rote for them. It’s an exploration usually.” The second was to reframe the activity so that it required students to consider alternative perspectives and think more deeply about what they are learning by asking them “to define concepts that somebody who didn’t already know the answer would understand.” By prompting students to consider a perspective different than their own, Chris primed the cognitive demand he was expecting of his students.

Michelle also explained how she plans for group work. Earlier, we described how Michelle developed a routine with her grouping strategies in order to encourage peer interactions. Not only did Michelle demonstrate deliberate forethought into the physical positioning of students and who they might interact with, she was also conscientious in establishing this grouping technique as a routine from the early days in the semester. Michelle mentioned another activity structure that necessitated peer interaction: “a round robin sort of thing where each team would have to put up their free body diagram on the board and the class would critique it.”

Although these examples demonstrate deliberate design of activities for participation, they do not necessarily call for a complete overhaul of the course. For instance, Betsy commented how instructors can facilitate active learning by making efforts to simply plan for smaller aspects of the course that might lend itself more to non-traditional teaching methods. She reflected, “It’d be important for faculty members to understand that you can actually do it in little pieces that you kind of stick into the course without really changing the course very much.”

#### Use incremental steps

Another new facilitation strategy, which was described by multiple instructors, was to “use to incremental steps,” which is also often referred to as “scaffolding” in the learning sciences literature. In Vygotskyian constructivist theory (Vygotsky 1978), “scaffolding” is

An instructional method whereby a teacher models the concept or skill to be learned, leads students through guided practice activities, and then offers various levels of teacher support while students practice the concept or skill independently. The purpose is to create a learning environment where students build confidence while mastering new skills or concepts. (Sullivan 2009)

Scaffolding, then, is when an instructor breaks down a complex cognitive task and provides supports to help students achieve the task, gradually removing these supports as students are better able to handle more complex steps on their own. In the interview data, scaffolding, or the use of incremental steps, can be seen in Timothy’s use of “…more hints, maybe more fill-ins and key gaps in the material before they start.” Another illustration of this strategy comes from Michelle:

What I ask them to focus on while they’re in the classroom is giving the problem set up, getting it to the point where it’s just math after that. If they can get to the point where they know what they’re supposed to do, then we’re good. Then if we’re doing something else, like if I’m going through an example on the board, I might ask them, ‘OK write down what your first step is going to be.’

Michelle broke down the tasks into incremental steps for her students in multiple ways. First, by “setting up” the problem, she provided students with a crucial first step to help them solve the problem. She was also modeling how to think about the problem and how to formulate a mathematical equation or expression out of a qualitative problem. She also provided a visual support by writing it on the board. As illustrated by these examples, scaffolding can occur in multiple ways and scaffolds can be created by manipulating different resources such as time, materials, and interpersonal engagement.

## Discussion

Our interview data showed that participants employed a variety of strategies to reduce resistance to active learning. These strategies were consistent with our prior framing of two broad categories of explanation and facilitation strategies*.* However, four additional facilitation strategies for reducing student resistance emerged from the interviews. Comparison across the strategies reveals that (1) instructors use a variety of strategies and implement strategies in different ways, (2) strategies are often explicitly designed to increase students’ engagement in active learning, (3) strategies are often interrelated, and (4) strategies have a temporal component.

### Variety of strategies

Instructors described a variety of ways to implement each of the strategies. While some chose explicit approaches, others had more implicit ways of implementing a strategy. In explaining the purpose of the activities, for example, some instructors chose to directly tell students about the purpose of the activities (Michelle, Betsy, Timothy, Esther, Tracey) while others engaged students in reflection and discussion to help them discover the purpose of the activities (Daniel). A similar variety in implementation was true for the strategies of explaining course expectations and explaining activity expectations.

Instructors also employed a diverse array of techniques when using facilitation strategies. When inviting students to ask questions, for example, some instructors walked around the room to be more available for questions, others designed activities with the intention of eliciting questions from students, and still others remained silent to create space for students to ask questions. Similarly, some instructors used more implicit ways of getting student feedback. For example, Daniel paid attention to students’ body language as a way of gaining feedback from students.

Perhaps the strategy that produced the greatest variety of methods was approaching non-participating students. Here, instructors discussed using everything from physical proximity, private conversations, calling on individual students, calling on teams, and using humor as techniques to engage students who appeared to be non-participatory. A variety of implementation tactics were also observed in the other facilitation strategies of developing a routine, grading on participation, designing activities for participation, and using incremental steps/scaffolding. Instructors attended to various aspects of classroom environment (such as the physical layout, student group compositions, the activity structure, or time) and were cognizant of how they could alter these factors to effectively employ a strategy.

#### Learning environment

Instructors frequently noted that they used a specific strategy to create an encouraging environment for students to engage in active learning. Though this rationale was evident for almost all of the strategies, it was often explicitly stated in the instructors’ discussions on explaining course expectations, assuming an encouraging demeanor, inviting students to ask questions, and developing a routine. For example, both Daniel and Tracey discussed how inviting questions allowed them to communicate that their classroom was an environment that embraced interaction and engagement and one where student voices were valued. Similarly, Michelle, William, Emily, and George reflected on how they tried to convey a demeanor of approachability and make students feel safe. Promoting a non-threatening environment for students to make mistakes and engage in active learning was also apparent in some of the techniques instructors used to approach non-participants. Likewise, much of the rationale for developing a routine was to “set the tone” and build a sense that students were expected to participate meaningfully throughout class. By employing these different strategies, the instructors ultimately sought to establish a learning environment that encouraged active engagement by the students.

#### Interrelatedness of the strategies

There was a great deal of interrelatedness across the strategies. For example, “walking around the room” was not only a strategy in itself but was pivotal in facilitating other strategies such as inviting questions, approaching non-participants, or assuming an encouraging demeanor. By creating physical proximity, instructors created opportunities for different kinds of interactions with students. As a result, students felt less threatened and more inclined to ask questions or participate in the activities, as noted by Samantha.

This interrelatedness is also seen across the explanation strategies. While an instructor is explaining the purpose of an activity, he or she might also explain what students are expected to do for the activity. Explanation strategies were also occasionally interrelated with facilitation strategies. For example, walking around the room allowed instructors to reinforce several of the explanation strategies. Similarly, in taking time to explain the purpose, course expectations, or activity directions, the instructor could assume a demeanor that may be encouraging and approachable to students.

#### Temporal nature of the strategies

The interviews illustrated a temporal aspect of strategy implementation. Some strategies were implemented during a class period, often somewhat spontaneously. Others occurred outside of class time, while still others lasted throughout the course term. Many of the strategies occurred during the class period, such as explain the purpose of the activities, explain course expectations, explain activity expectations, walk around the room, approach non-participants, and invite questions. On the other hand, designing activities for participation required some pre-emptive planning and therefore usually occurred before class. Strategies like developing a routine and assuming an encouraging demeanor seemed to have a more continuous implementation in that these strategies needed to be enacted consistently throughout the course term in order to be effective. Other strategies like soliciting student feedback occurred both in and out of class. Thus, in considering ways to facilitate active learning, it is important to recognize that not all strategies must occur within class or within a single class period.

#### Comparison with prior work

Most of the strategies described in this paper have been previously identified as best practices for teaching or implementing active learning (Armstrong 1998; Arum and Roksa 2011; Felder 2011; Felder and Brent 1996, 2010; Higbee and Burney 2011; Johnson 2003; Lake 2001; Michael 2007; Moffett and Hill 1997). The difference, and the main contribution of this paper, is that this is the first study that directly and empirically links the strategies to also reducing student resistance to active learning. The student survey results that accompany this interview data (Finelli, C. J., Nguyen, K., DeMonbrun, R.M., Borrego, M., Prince, M., Henderson, C., Husman, J: Reducing student resistance to active learning: Strategies for instructors, forthcoming) show that using these strategies resulted in greater participation from students, less distraction, and more positive course evaluations. Further, facilitation strategies had a stronger influence on these outcomes than explanation strategies.

However, there were some differences between student and instructor perceptions of the strategies. Students reported noticing explanation strategies being used more often than facilitation strategies. Although this qualitative data does not address frequency, a comparison of instructor reported means for explanation and facilitation strategies suggested that instructors utilized facilitation strategies more often than explanation strategies. Future work might better elucidate these relationships and compare the differences between what students and faculty perceive and report. Finally, the instructors here recommend developing a routine as one of the strategies. They describe setting a tone for the class in which students expect to participate every class period, sometimes through a specific structure such as a worksheet or assigned group. However, we note that in some of our observation work (Shekhar et al., 2015), students can become bored with a repetitive active learning format such as think-pair-share, and they are more likely to participate when there is more variety among the activities. It is possible, of course, to both set a tone for consistent participation and use a variety of activities and assignments.

#### Practical implications

The findings of this study give rise to several practical implications, particularly as they concern instructors seeking to increase their use of active learning. Perhaps the most important implication is to emphasize the numerous ways that instructors might employ explanation and facilitation strategies. All instructors are different with varied strengths, styles and instructional contexts; there is no “one size fits all” approach for making active learning strategies work. The most successful efforts to diffuse educational innovations recognize this. Montfort et al. (2012), for example, found that the most important factor influencing instructors’ decisions to adopt evidence-based instructional practices was their perception of how well it would work in the context of their own course. Providing several methods for implementing explanation and facilitation strategies, as is done here, increases the odds that instructors will identify methods that they believe will work for them, which should increase the use of these strategies in practice.

The temporal nature of instructional strategies further broadens these options for both instructors and faculty development personnel. Rather than focusing exclusively on strategies that can be implemented in the classroom, these findings emphasize the importance of course planning. A crucial and productive instructional design question to ask before implementing a new instructional method is, “What could go wrong?” Encouraging instructors to think in advance about how students might resist active learning strategies and how they can structure the class activities to minimize these problems is sound, practical advice. Faculty development personnel should encourage instructors to engage in this exercise.

Finally, our findings emphasize the wider importance of classroom climate in influencing how students respond to active learning strategies. Resistance is often defined in terms of student behaviors (e.g., non-participation in activities, complaints about the activity, giving low course evaluations), but these behaviors are likely mediated by students’ emotions and attitudes. The importance of creating a supportive learning environment, both through what is said and what is communicated in other ways, is seen in several instructors’ interviews. It follows then that engineering instructors should be cognizant of the personal and affective components essential for the successful implementation of active learning strategies.

## Limitations

This study has a few limitations to note. Instructors self-identified as using active learning in their classes. We triangulated these reports with additional data sources (surveys and interview questions). Some level of active learning is required for a study of resistance to active learning; otherwise, there is nothing for students to potentially resist. Yet, the instructors who volunteered to have their teaching studied in such detail were particularly experienced and confident about their use of active learning. The findings should be taken as the experiences and professional opinions of expert rather than typical engineering instructors. The participant interview protocol focused on strategies from the literature and our prior classroom observations and perhaps biased the discussion. Nonetheless, this study still identified four additional strategies. Another limitation of this study was that the interviews relied on self-reported data. While we triangulated information from students and their instructors, the interview data still possess the risk of having been potentially biased by selective memory, perceptions of positive outcomes in the classroom as a result of instructors’ own actions, or exaggeration in their use of the strategies. Nonetheless, a majority of the strategies incorporated into the interview protocol were first identified based on results from student surveys and prior research. Though it was beyond the scope of this study, future work that directly compares use of these strategies to student learning, particularly through comparison to uncover increases in student learning gains, could provide stronger evidence of the efficacy of these strategies.

Finally, another potential limitation of this study was that the sample of instructor participants mostly represented members of dominant culture groups, potentially reinforcing stereotypes of who the typical engineering instructor or engineer is. However, since the instructors self-identified and volunteered to be participants in our study, it was difficult to control for the over-representation of one demographic group. That said, it is nevertheless encouraging that just under half of the 18 instructors interviewed were female engineering faculty, potentially subverting some of the predominant gender stereotypes of who occupies these roles.

## Conclusions

Prior undergraduate STEM education research has in large part focused on the efficacy of active learning without paying much empirical attention to the ways instructors can reduce anticipated student resistance to active learning. This study aims to fill that gap and provides a resource of empirically derived strategies that instructors can use to encourage student participation in active learning. Our prior analyses show that students’ recollection of their instructors’ use of explanation and facilitation strategies correlate with increased participation, lower levels of distraction, and more positive course evaluations. These strategies included explaining course expectations, explaining the purpose of the activity, explaining activity expectations, walking around the room, assuming an encouraging demeanor, inviting student questions, soliciting student feedback, and approaching non-participating students. In addition to corroborating instructors’ use of these strategies, this study also identified four new strategies for promoting student engagement with active learning: developing a routine, grading on participation, conscientiously designing activities for student-participation, and using incremental activities. These strategies provide concrete, actionable ways for instructors to reduce student resistance to active learning. The findings demonstrate that there are many possible ways for instructors to encourage participation, reduce distraction and potentially increase evaluation scores while being consistent with their beliefs and preferences. Several participants demonstrated forethought in their use of these various strategies. Instructors should therefore be reflective in deciding which strategies would best fit their intended instructional goals and classroom scenarios and why.

Although not explored in this study, another compelling theme apparent in the interview data warrants further examination, namely the influence of instructors’ beliefs about teaching and learning on instructional decision-making and practices, particularly in the implementation and facilitation of active learning. The findings of such work will help identify more targeted ways to assist instructors in their efforts to adopt active learning instructional practices and consequently promote widespread instructional change (Pelch and McConnell 2016; Lund and Stains 2015). Other future works in this area should strive to study the instructional practices of a more demographically diverse sample of instructors, so as to not only challenge dominant narratives but to potentially uncover other important and innovative strategies that may be lacking representation in the extant literature.

## References

[CR1] Arce, P. (1994). The colloquial approach: an active learning technique. *Journal of Science Education and Technology*, *3*(3).

[CR2] Armbruster P, Patel M, Johnson E, Weiss M (2009). Active learning and student-centered pedagogy improve student attitudes and performance in introductory biology. CBE-Life Sciences Education.

[CR3] Armstrong JS (1998). Are student ratings of instruction useful?. American Psychologist.

[CR4] Arum R, Roksa J (2011). Academically adrift.

[CR5] Bacon D, Stewart K, Silver W (1999). Lessons from the best and worst student team experiences: how a teacher can make the difference. Journal of Management Education.

[CR6] Bentley FJB, Kennedy S, Semsar K (2011). How not to lose your students with concept maps. Journal of College Science Teaching.

[CR7] Boyatzis RE (1998). Transforming qualitative information: thematic analysis and code development. The SAGE glossary of the social and behavioral sciences.

[CR8] Brent R, Felder RM (2009). Analysis of fifteen years of the National Effective Teaching Institute.

[CR9] Carlson K, Winquist J (2011). Evaluating an active learning approach to teaching introductory statistics: a classroom workbook approach. Journal of Statistics Education.

[CR10] Chi MT, Wylie R (2014). The ICAP framework: linking cognitive engagement to active learning outcomes. Educational Psychologist.

[CR11] Council, N. R (2012). Discipline-based educational research: understanding and improving learning in undergraduate science and engineering.

[CR12] Dancy MH, Henderson C (2012). Experiences of new faculty implementing research-based instructional strategies.

[CR13] DeMonbrun, R.M., Borrego, M., Finelli, C. J., Prince, M., Henderson, C., & Waters, C. (April 2017). Creating an instrument to measure student response to instructional practices. Journal of Engineering Education, 106(2).

[CR14] Donohue S, Richards L (2009). Factors affecting student attitudes towards active learning activities in a graduate engineering statistics course.

[CR15] Education ASOE (2012). Innovation with impact: creating a culture for scholarly and systematic innovation in engineering education.

[CR16] Felder, RM. (1992). How about a quick one? *Chemical Engineering Education*, *26*(1).

[CR17] Felder RM (1994). Any questions?. Chemical Engineering Education.

[CR18] Felder RM (2011). Hang in there: dealing with student resistance to learner-centered teaching. Chemical Engineering Education.

[CR19] Felder RM, Brent R (1996). Navigating the bumpy roa to student-centered instruction. College Teachin.

[CR20] Felder, RM, & Brent, R. (2009). Active learning: an introduction. *ASQ Higher Education Brief*, *2*(4).

[CR21] Felder RM, Brent R (2010). The National Effective Teaching Institute: assessment of impact and implications for faculty development. Journal of College Science Teaching.

[CR22] Finelli, C. J., Daly, S. R., & Richardon, K. M. (2014). Bridging the research-to-practice gap: Designing an institutional change plan using local evidence. Journal of Engineering Education, 103(2), 331–361.

[CR23] Freeman S, Eddy SL, McDonough M, Smith MK, Okoraoafor N, Jordt H, Wenderoth MP (2014). Active learning increases student performance in science, engineering, and mathematics. Proceedings of the National Academy of Sciences.

[CR24] Friedrich K, Sellers S, J B, Nilson DRL (2009). Thawing the chilly climate: inclusive teaching resources for science, technology, engineering, and math. To improve the academy: resources for faculty, instructional, and organizational development.

[CR26] Froyd J, Borrego M, Cutler S, Henderson C, Prince M (2013). Estimates of use of research-based instructional strategies in core electrical or computer engineering courses. IEEE Transactions on Education.

[CR27] Goodwin, L, Miller, JE, Cheetham, RD. (1991). Teaching freshman to think: does active learning work? *Biosciences*, *41*(719–722).

[CR28] Hall SR, Waitz I, Brodeur DR, Soderholm DH, Nasr R (2002). Adoption of active learning in a lecture-based engineering class.

[CR29] Henderson, C, & Dancy, MH. (2009). The impact of physics education research on the teaching of introductory quantitative physics in the United States. *Physical Review Special Topics: Physics Education Research*, *5*(2).

[CR30] Higbee M, Burney JM (2011). Facing the elephant: overcoming faculty fears of active learning. Paper presented at the Network for Academic Renewal Conference. archive.aacu.org/meetings/generaleducation/gened2011/documents/CS49.ppt

[CR31] Hoffman, E. (2001). Successful application of active learning techniques to introductory microbiology. *Microbiology Education*, *2*(1). 10.1128/me.2.1.5-11.2001 PMC3633112 23653538

[CR32] Hora MT, Ferrare JJ, Oleson A (2012). Findings from classroom observations of 58 math and science faculty.

[CR33] Johnson VE (2003). Grade inflation: a crisis in college education.

[CR34] Kvam P (2000). The effect of active learning methods on student retention in engineering statistics. *The Amerian Statistician*, *54*(2), 136–140.

[CR35] Lake D (2001). Student performance and perceptions of a lecture-based course compared with the same course utilizing group discussion. Physical Therapy.

[CR36] Leckie, R. M. (2001). Engage students with active learning strategies. Retrieved from serc.carleton.edu/NAGTWorkshops/intro/activelearning.html

[CR37] Lund TJ, Stains M (2015). The importance of context: an exploration of factors influencing the adoption of student-centered teaching among chemistry, biology, and physics faculty. International Journal of STEM Education.

[CR38] Michael J (2006). Where’s the evidence that active learning works?. Advances in Physiology Education.

[CR39] Michael J (2007). Faculty perceptions about barriers to active learning. College Teaching.

[CR40] Moffett BS, Hill KB (1997). The transition to active learning: a lived experience. Nurse Educator.

[CR41] Montfort D, Brown S, Pegg J (2012). The adoption of a capstone assessment instrument. Journal of Engineering Education.

[CR42] Nguyen, K., DeMonbrun, R.M., Borrego, M., Prince, M., Husman, J., Finelli, C. J., Waters, C. (2017a). The variation of nontraditional teaching methods across 17 undergraduate engineering. classrooms. Paper presented at the 2017American Society of Engineering Education Annual Meeting, Columbus, OH.

[CR43] Nguyen, K., Husman, J., Borrego, M., Shekhar, P., Prince, M., Finelli, C. J., Waters, C. (2017b). Students’ expectations, types of instruction, and instructor strategies predicting student response to active learning. International Journal of Engineering Education, 33(1).

[CR44] Nguyen, K. A., DeMonbrun, M., Borrego, M., Husman, J., Prince, M., Finelli, C., Waters, C. (2017c). The tensions measuring instructional practices. In 2017 Research in Engineering Education Symposium, REES 2017 Research in Engineering Education Network.

[CR45] O’Brocta, R, & Swigart, S. (2013). Student perceptions of a top 200 mediation course utilizing active learning techniques. *Currents in Pharamacy Teaching and Learning*, *5*.

[CR46] Oakley BA, Hanna DM, Kuzmyn A, Felder RM (2007). Best practices involving teamwork in the classroom: results from a survey of 6435 engineering student respondents. IEEE Transactions on Education.

[CR47] Pelch MA, McConnell DA (2016). Challenging instructors to change: a mixed methods investigation on the effects of material development on the pedagogical beliefs of geoscience instructors. International Journal of STEM Education.

[CR48] Prince M (2004). Does active learning work? A Review of the Research. Journal of Engineering Education.

[CR49] Prince, M., Borrego, M., Henderson, C., Cutler, S., & Froyd, J. (2013). Use of research-based instructional strategies in core chemical engineering courses. Chemical Engineering Education, 47(1), 27–37.

[CR50] Prince, M., & Felder, R. M. (2007). The many faces of inductive teaching and learning. Journal of College Science Teaching, 36(5).

[CR51] Rangachari P (1991). Design of a problem-based undergraduate course in pharmacology: implications for the teaching of physiology. The American Journal of Physiology.

[CR52] Reddy IK (2000). Implementation of a pharmaceutics course in a large class through active learning using quick-thinks and case-based learning. American Journal of Pharmaceutical Education.

[CR53] Richardson, D, & Birge, B. (1995). Teaching physiology by combined passive (pedagogical) and active (andragogical) methods. *Advances in Physiology Education*, *13*(1). 10.1152/advances.1995.268.6.S66 7598176

[CR54] Shadle SE, Marker A, Earl B (2017). Faculty drivers and barriers: laying the groundwork for undergraduate STEM education reform in academic departments. International Journal of STEM Education.

[CR55] Shekhar P, DeMonbrun RM, Borrego M, Finelli CJ, Prince M, Henderson C, Waters C (2015). Development of an observation protocol to study undergraduate engineering student resistance to active learning. International Journal of Engineering Education.

[CR56] Strobel J, van Barneveld A (2009). When is PBL more effective? A meta-synthesis of meta-analyses comparing PBL to conventional classrooms. Interdisciplinary Journal of Problem-based Learning.

[CR57] Sullivan LE (2009). Scaffolding. The SAGE Glossary of the Social and Behavioral Sciences (Vol. 3, pp. 460).

[CR58] Vygotsky LS, Cole M, John-Steiner V, Scribner S, Souberman E (1978). Mind in society: the development of higher psychological processes.

[CR59] Wilke RR (2003). The effect of active learning on student characteristics in human physiology course for non majors. Advances in Physiology Education.

[CR60] Yadav A, Lundeberg M, Subedi S, Bunting C (2011). Problem-based learning in an undergraduate electrical engineering course. Journal of Engineering Education.

